# Supplementary material for: Efficacy and Safety of Clonidine in the Treatment of Acute Mania in Bipolar Disorder: A Systematic Review
Source: Brain Sci. 2023 Mar 25;13(4):547. doi: 10.3390/brainsci13040547 (PMC10137105; doi:10.3390/brainsci13040547)
Supplement: Supplementary file 1 [file brainsci-13-00547-s001.zip › brainsci-2306422-supplementary.pdf]

Supplemental Data File

Table S1: Search Strategy

Clonidine in the treatment of acute mania

OVID

Database(s): **Ovid MEDLINE(R) 1946 to Present and Epub Ahead of Print, In-Process & Other Non-Indexed Citations and Ovid MEDLINE(R) Daily, EBM Reviews - Cochrane Central Register of Controlled Trials** December 2022**, EBM Reviews - Cochrane Database of Systematic Reviews** 2005 to January 18, 2023**, Embase** 1974 to 2023 January 19

Search Strategy:

#	Searches
1	Clonidine/
2	(clonidine or catapress* or catasan or chlofazolin or chlophazolin or chlophelin or clinidine or clofelin* or clomidine or clondine or clonicel or clonidin or clonipresan or clonistada or clonnirit or clophelin or clopheline or daipres or dixarit or duraclon or haemiton or hemiton or hypodine or isoglaucon or jenloga or kapvay or normopresan or normopresin or paracefan or sulmidine or taitecin).ti,ab.
3	Receptors, Adrenergic, alpha-2/ or alpha 2 adrenergic receptor/
4	("alpha2-adrenoceptor*" or "alpha-2-adrenoceptor*" or "alpha2-adrenoreceptor*" or "alpha-2-adrenoreceptor*" or "alpha2-noradrenergic-receptor*" or "alpha2-adrenergic-receptor*").ti,ab.
5	1 or 2 or 3 or 4
6	exp bipolar disorder/
7	(mania or manic or bipolar or hypomanic or hypomania or cyclothymia).ti,ab.
8	6 or 7
9	5 and 8
10	remove duplicates from 9

SCOPUS

1	TITLE-ABS-KEY (clonidine OR catapress* OR catasan OR chlofazolin OR chlophazolin OR chlophelin OR clinidine OR clofelin* OR clomidine OR clondine OR clonicel OR clonidin OR clonipresan OR clonistada OR clonnirit OR clophelin OR clopheline OR daipres OR dixarit OR duraclon OR haemiton OR hemiton OR hypodine OR isoglaucon OR jenloga OR kapvay OR normopresan OR normopresin OR paracefan OR sulmidine OR taitecin) OR TITLE-ABS-KEY ("alpha2-adrenoceptor*" OR "alpha-2-adrenoceptor*" OR

	"alpha2-adrenoreceptor*" OR "alpha-2-adrenoreceptor*" OR "alpha2-noradrenergic-receptor*" OR "alpha2-adrenergic-receptor*")
2	TITLE-ABS-KEY (mania OR manic OR bipolar OR hypomanic OR hypomania OR cyclothymia)
3	1 AND 2
4	INDEX(embase) OR INDEX(medline) OR PMID(0* OR 1* OR 2* OR 3* OR 4* OR 5* OR 6* OR 7* OR 8* OR 9*)
5	3 AND NOT 4
6	DOCTYPE(ed) OR DOCTYPE(bk) OR DOCTYPE(er) OR DOCTYPE(no) OR DOCTYPE(sh) OR DOCTYPE(ch)
**7**	5 AND NOT 6

Web of Science

1	TS=(mania OR manic OR bipolar OR hypomanic OR hypomania OR cyclothymia)
2	TS=(clonidine OR catapress* OR catasan OR chlofazolin OR chlophazolin OR chlophelin OR clinidine OR clofelin* OR clomidine OR clondine OR clonicel OR clonidin OR clonipresan OR clonistada OR clonnirit OR clophelin OR clopheline OR daipres OR dixarit OR duraclon OR haemiton OR hemiton OR hypodine OR isoglaucon OR jenloga OR kapvay OR normopresan OR normopresin OR paracefan OR sulmidine OR taitecin OR "alpha2-adrenoceptor*" OR "alpha-2-adrenoceptor*" OR "alpha2-adrenoreceptor*" OR "alpha-2-adrenoreceptor*" OR "alpha2-noradrenergic-receptor*" OR "alpha2-adrenergic-receptor*")
3	1 AND 2
4	PMID=(0* or 1* or 2* or 3* or 4* or 5* or 6* or 7* or 8* or 9*)
5	3 NOT 4
6	5 Refined By:Document Types: Article

Table S2A. Risk of bias for studies included in the systematic review.

Criteria	Ahmadpanah et al., 2022	Janicak et al., 1989	Hardy et al., 1989	Giannini et al., 1986	Giannini et al., 1985
**Random sequence generation (selection bias)**	Low Risk	Unclear risk	Low risk	Unclear risk	Unclear risk
**Allocation concealment (selection bias)**	High Risk	Unclear risk	Unclear risk	Unclear risk	Unclear risk
**Blinding of participants and personnel**	Low Risk	Low Risk	Low risk	Low risk	Low risk
**Blinding of outcome assessment (detection bias)**	Low Risk	Low Risk	Low risk	Low risk	Low risk
**Incomplete outcome data addressed (attrition bias)**	Low Risk	High Risk	Unclear	High risk	High Risk
**Selective reporting (reporting bias)**	Low Risk	High Risk	Low risk	Low risk	Low risk
**Other bias**	Unclear risk	Unclear risk	Unclear risk	Unclear risk	Unclear risk

Table S2B. Quality assessment of the non-randomized studies using the Methodological Index for Non-Randomized Studies (MINORS)

**Methodological items for non-randomized studies**		**Jouvent et al., 1980**	**Tudorache, and Diacicov, et al., 1991**	**Hardy et al., 1986**	**Giannini et al., 1983**
1	A clearly stated aim: the question addressed should be precise and relevant in the light of available literature	2	1	2	2
2	Inclusion of consecutive patients: all patients potentially fit for inclusion (satisfying the criteria for inclusion) have been included in the study during the study period (no exclusion or details about the reasons for exclusion)	1	1	1	1
3	Prospective collection of data: data were collected according to a protocol established before the beginning of the study	0	0	2	2
4	Endpoints appropriate to the aim of the study: the unambiguous explanation of the criteria used to evaluate the main outcome which should be in accordance with the question addressed by the study. Also, the endpoints should be assessed on an intention-to-treat basis.	1	2	2	2
5	Unbiased assessment of the study endpoint: blind evaluation of objective endpoints and double-blind evaluation of subjective endpoints. Otherwise, the reasons for not blinding should be stated	1	2	0	1
6	Follow-up period appropriate to the aim of the study: the follow-up should be sufficiently long to allow the assessment of the main endpoint and possible adverse events	1	2	1	1
7	Loss to follow-up less than 5%: all patients should be included in the follow-up. Otherwise, the proportion lost to follow up should not exceed the proportion experiencing the major endpoint	2	2	2	2
8	Prospective calculation of the study size: information of the size of the detectable difference of interest with a calculation of 95% confidence interval, according to the expected incidence of the outcome event, and information about the level for statistical significance and estimates of power when comparing the outcomes	0	0	0	0

†The items are scored 0 (not reported), 1 (reported but inadequate), or 2 (reported and adequate). The global ideal score being 16 for non-comparative studies.
